# The potential of neutrophil gelatinase-associated lipocalin in management of acute kidney injury and peritoneal dialysis-related peritonitis: a narrative review

**DOI:** 10.3389/fneph.2025.1749827

**Published:** 2026-01-27

**Authors:** Xiao Fu, Yiting Shu, Yun Zhang

**Affiliations:** Global Medical Information and Education, Fresenius Medical Care Asia Pacific Limited, Hong Kong, Hong Kong SAR, China

**Keywords:** acute kidney injury, biomarker, early detection, molecular form, neutrophil gelatinase-associated lipocalin, peritoneal dialysis-related peritonitis, point-of-care testing

## Abstract

Neutrophil gelatinase-associated lipocalin (NGAL) is a biomarker extensively studied in multiple diseases. While its application in chronic kidney disease (CKD) and kidney transplant patients is relatively limited, NGAL has shown significant promise in the early detection and diagnosis of acute kidney injury (AKI), which may improve more timely management and potentially better clinical outcomes. In addition, NGAL has demonstrated promising utility in identifying peritoneal dialysis-related peritonitis (PDRP) and monitoring the treatment response. This review aims to provide an in-depth overview of the available research findings of NGAL in the management of AKI and PDRP, having these two conditions discussed together is particularly important for nephrologists who manage both conditions, especially to explore the potential of more specific NGAL forms, such as monomer NGAL and homodimer NGAL, to enhance early diagnosis and effective management of AKI and PDRP.

## Highlights

- In AKI, NGAL enables early AKI detection within hours, outperforming serum creatinine, while also indicating AKI severity and predicting the need for KRT, though standardized cut-off values are still often lacking.
- Monomeric NGAL shows promise for kidney-specific injury differentiation in AKI, pending further validation to enable more specific early diagnosis.
- In PDRP, effluent NGAL demonstrates comparable or possibly superior diagnostic accuracy to WBC counts and some other markers.
- Homodimeric NGAL POCT offers high sensitivity/specificity for early peritonitis diagnosis; its application in home settings may shorten symptom-to-treatment time and potentially improve outcomes pending clinical validation.
- Form-specific assays may enhance NGAL’s precision by distinguishing renal vs. systemic origins and have the potential to improve clinical management of AKI and PDRP after more validation.

## 1 Introduction

Neutrophil Gelatinase-Associated Lipocalin (NGAL), a multifunctional protein initially identified in activated neutrophils, regulates iron homeostasis and modulates inflammation. NGAL can chelate siderophores synthesized by bacteria, thereby preventing bacterial iron acquisition and inhibiting bacterial growth and proliferation (1). Although NGAL was initially identified in human neutrophils, many other sources were discovered later to secrete NGAL too, including the kidneys, liver, and epithelial cells (1).

Different molecular forms of NGAL, including 25-kD monomer, 45-kD homodimer, and 135-kD heterodimer (formed when NGAL is covalently conjugated with matrix metalloproteinase-9, MMP-9), indicate different sources and have different clinical significance (2). Monomeric NGAL is predominantly released by kidney tubular epithelial cells (to a lesser extent the heterodimer as well), making monomeric NGAL most relevant for kidney injury. In contrast, neutrophils synthesize and release all NGAL forms, with the homodimer being the predominant form during inflammation, especially bacterial infections (2–5). A comparative summary of NGAL molecular forms, their sources, and clinical relevance is provided in **Table 1**.

**Table 1:** Molecular forms of NGAL, their predominant sources, and clinical relevance.

NGAL molecular forms	Approximate molecular weight	Predominant cellular sources	Clinical relevance
Monomer	~ 25 kDa	Kidney tubular epithelial cells	Kidney−derived signal; enriched in acute kidney injury (AKI) for example as early biomarker
Homodimer	~ 45 kDa	Activated neutrophils	Neutrophil activation, enriched mainly in infection, inflammation
Heterodimer	~ 135 kDa	Neutrophils, lower abundance in kidney related samples	Probably more in chronic disease progression, including cancer

NGAL is an acute-phase protein involved in inflammation, with its serum concentrations showing a positive correlation with inflammatory cytokines (1). In addition, it is also involved in various clinical conditions. In oncology, elevated NGAL expression is linked to tumor aggressiveness and metastasis in breast, prostate, gastric, and other cancers (6–11). In cardiovascular disease, NGAL is expressed in heart tissue, including cardiomyocytes, vascular wall cells, fibroblastoid cells, and atherosclerotic plaques, with elevated plasma levels observed in coronary artery disease as well as in both acute and chronic heart failure (12–14). Moreover, NGAL may play a potential role in the pathogenesis of cognitive impairment, Alzheimer’s disease, and chronic obstructive pulmonary disease (15–17).

In nephrology, NGAL levels rise in kidney dysfunction, aiding early detection of CKD and predicting its progression, mortality, and complications. It serves as a sensitive marker for IgA nephropathy, systemic lupus erythematosus (SLE), diabetic nephropathy, and autosomal dominant polycystic kidney disease (ADPKD) (18–22). In kidney transplantation, NGAL reflects graft function and predicts acute rejection, with elevated levels often preceding rejection events (23–25).

In particular, NGAL has demonstrated significant potential in diagnosing AKI, assessing risk, predicting its occurrence and severity, and monitoring kidney recovery, supporting its consideration for clinical application (26). For peritoneal dialysis-related peritonitis (PDRP), even though its clinical use is far less established, some studies have already found a positive correlation between NGAL levels and white blood cell (WBC) counts in peritoneal effluent, suggesting NGAL can serve as a marker to diagnose peritonitis and monitor the response to treatment too (27–29). Although AKI and PDRP are distinct clinical conditions, they share a common need for rapid recognition and timely intervention. NGAL provides a unifying perspective through its form-specific roles: monomeric NGAL reflects tubular injury in AKI, while homodimeric NGAL indicates neutrophil-driven inflammation in PDRP. This dual relevance makes NGAL particularly important for nephrologists, who frequently manage both conditions.

However, the common use of total NGAL assays, which measure all forms collectively, may limit its potential in disease management due to its possible insufficient specificity, especially for AKI. While its clinical use in other areas is relatively constrained, this review will focus on its application in the two kidney conditions, AKI and PDRP, its current utilization, and the potential of more specific NGAL forms, such as monomeric NGAL and homodimer NGAL. The review will discuss the limitations of the current use of (total) NGAL and the potential benefits of employing more specific NGAL forms in the management of kidney diseases, particularly in diagnosis.

## 2 Methods

This narrative review synthesized evidence on NGAL, with a primary focus on its role in AKI and PDRP. The literature search was conducted primarily in PubMed and Google Scholar up to November 2025, applying combinations of keywords such as “neutrophil gelatinase-associated lipocalin”, “NGAL”, “acute kidney injury”, “AKI”, “peritoneal dialysis”, and “peritonitis”, including specific forms like “homodimer NGAL” and “monomer NGAL”. Boolean operators (AND/OR) were applied to optimize search relevance. For NGAL in AKI, given the extensive literature available, we focused on more recent or seminal original studies that were relevant to our main focus areas. For NGAL in PDRP, where literature was limited, we aimed for comprehensive coverage of all available studies, supplemented by manual screening of reference lists and attention to recent key publications. Preclinical studies were also considered when human data were limited or absent. While original research was prioritized, high-quality reviews, relevant clinical guidelines, and consensus statements were included to provide context, summarize established evidence, highlight research gaps, and ensure alignment with current clinical practice. Inclusion criteria were primarily English-language articles reporting NGAL in AKI or PDRP; exclusion criteria were articles on unrelated topics or those with insufficient data.

## 3 NGAL in AKI

AKI is a broad clinical syndrome characterized by an abrupt decrease in kidney function, encompassing specific renal diseases (e.g., acute interstitial nephritis, acute glomerulonephritis, vasculitic renal diseases), non-specific insults (e.g., ischemia, nephrotoxic injury), and extrarenal etiologies (e.g., prerenal azotemia and acute postrenal obstructive nephropathy) (30). The current diagnosis of AKI is based on changes in serum creatinine (SCr) and urine output, following the Kidney Disease: Improving Global Outcomes (KDIGO) guidelines (30), which may not be sensitive enough to detect early-stage AKI, especially in the stage of kidney damage but not yet reflected by function markers. NGAL has shown potential as a damage biomarker for AKI, as its levels increase significantly and quickly in response to kidney injury.

NGAL rises within hours of renal tubular injury—significantly earlier than serum creatinine, which typically begins to increase roughly after 2 days. NGAL elevation has been associated with greater AKI severity, delayed renal recovery, higher mortality, and increased need for kidney replacement therapy; when integrated with current AKI staging, it enhances timely risk stratification, informs clinical decision-making, and may contribute to improved outcomes.

Although the KDIGO guideline published in 2012 recognizes NGAL as a kidney damage marker in early detection and reports clinical data on its prognostic utility, e.g. predicting kidney replacement therapy (KRT) requirement, it does not incorporate NGAL or other biomarkers into its diagnostic criteria or recommendations for clinical intervention. This reflects not only the limited data available at that time but also widely recognized challenges, including heterogeneous evidence, lack of standardized cut-offs and assay harmonization, and insufficient proof that biomarker-guided care improves patient-centered outcomes.

While the updated KDIGO AKI guideline is still in development, the expert consensus (Acute Disease Quality Initiative, ADQI) has already proposed in 2020 incorporating biomarkers into AKI staging and recognized NGAL’s value for diagnosing AKI and stratifying its severity (26). It advocates the combined use of damage and functional biomarkers—including NGAL—for early diagnosis, risk assessment, and prognostic evaluation.

However, apart from the lack of standardized cut-offs and assay harmonization, the specificity of total NGAL assays may remain limited. Evaluating more specific NGAL molecular forms has the potential to further improve its accuracy and specificity of AKI diagnosis, prediction of AKI severity and KRT initiation. The following sections will provide detailed data on NGAL’s capability to function as a diagnostic, risk stratification, and prognostic tool, as well as the potential advantages of the more specific NGAL form.

### 3.1 NGAL’s role in early diagnosis of AKI

According to the evidence from the past decades, NGAL has been recognized as a potential early-stage biomarker to detect AKI compared to the traditional biomarkers such as SCr, which rises rather late. Studies show that SCr reaches the AKI diagnosis criteria in 2–3 days after the kidney injury initiation in cardiac surgery patients and peak on or after day 3 of surgery in nearly one-fifth of patients with severe AKI, and nearly half among which had doubled SCr lasted for 2 days or longer despite timely treatment (31, 32), while elevated NGAL levels measured at 2–6 hours after surgery may predict subsequent AKI, with the area under the receiver-operating characteristic curve (AUC) of 0.67 for urine NGAL (uNGAL) and 0.7 for plasma NGAL (pNGAL) at 6 hours (31), 0.702 for uNGAL at 4 hours (33), and 0.998 for uNGAL at 2 hours (34). Both serum NGAL and uNGAL levels remained elevated during active renal injury but decline rapidly within 48 hours once the injury is resolving after treatment in patients with septic associated AKI, and a smaller decline at 48 hours was associated with higher CKD risk (35).

In an acute heart failure setting, NGAL appears to be a more rational indicator to predict early AKI during hospitalization compared to B-type natriuretic peptide (BNP) and cystatin C, with a sensitivity 85%, a specificity 80%, and an AUC of 0.81. Notably, NGAL is also the only biomarker among three that is capable of identifying patients who subsequently developed AKI during their hospital stay (36). Both uNGAL and serum NGAL were significant predictors of AKI in critically ill ICU patients, accurately forecasting AKI development within 3–7 days, whereas serum cystatin C showed no predictive value (37). uNGAL is also suggested to be a more specific predictor of early AKI compared to IL-18 with a pooled effect size of 1.09 (95% CI 1.03–1.15, P = .004) in specificity (38).

However, the lack of well-characterized NGAL cut-off value in AKI diagnosis limits its clinical application and therefore requires standardization in further studies (39). Current cut-offs of uNGAL and pNGAL in different studies vary between 78.2–247 ng/ml and 150–417 ng/ml, respectively, in terms of different clinical settings and comorbidities (40). Meanwhile, NGAL levels also increase in other chronic and acute inflammatory conditions encountered in the ICU, such as sepsis, CKDs, and urinary tract infections (UTIs), reducing its specificity as a clinical diagnostic marker for AKI (41).

Wu et al. suggested that monomeric uNGAL is likely associated with kidney injury in cats; they found that the proportion of cats with monomeric uNGAL is higher in the AKI group compared to the CKD group, indicating greater severity of kidney injury in AKI (42). Notably, they also found distinct patterns emerged for NGAL molecular forms within the CKD group: urinary NGAL monomer was detected in a significantly higher percentage of cats in CKD stages 3 and 4 compared to control and CKD stage 2 cats; homodimeric NGAL showed no association; and heterodimeric NGAL exhibited a non-significant trend towards association with CKD stage. Meanwhile, Hsu et al. found that monomeric uNGAL is more frequently detected in dogs with renal diseases than those without and is significantly correlated with abnormal blood urea nitrogen (BUN) and SCr levels, reflecting its association with kidney injury (43). In multiple myeloma patients, Woziwodzka et al. found that uNGAL monomer was interrelated with other urinary biomarkers of tubular injury and estimated lower glomerular filtration rate and believed that it might be a useful biomarker for identifying tubular renal damage (44). By analyzing urine samples collected from intensive care units, Mårtensson et al. devised a ratio of enzyme-linked immunosorbent assay ELISA-1/ELISA-2 to amplify the monomeric NGAL signal, which increased significantly during AKI development (45).

Unfortunately, most studies utilizing monomeric NGAL for AKI diagnosis and detection did not directly compare it with other NGAL assays. In contrast, Mårtensson et al. clearly demonstrated that the monomeric NGAL signal significantly increased during AKI development, whereas other assays did not perform as effectively in reflecting the progression of kidney injury (45). Overall, even though there has been a clear signal, it may still be too early to definitively confirm the superiority of monomeric NGAL in diagnosing AKI. Eventually, it seems necessary to develop a test that specifically detects monomeric NGAL derived from the tubular epithelium, which would possess greater accuracy and sensitivity in identifying AKI (2).

### 3.2 NGAL’s role in differential diagnosis of AKI

AKI arises from multifactorial etiologies. Sepsis is considered the most common one, followed by major surgery, cardiogenic shock, hypovolemia, complications with medications, etc. (46, 47). Pathophysiologically, AKI can be categorized into 3 groups: prerenal (decreased renal perfusion), intrinsic (direct damage to the kidneys), and postrenal (urinary tract obstruction, such as by bladder obstruction or urinary stones) (48, 49). The current standard diagnostic tool for AKI, SCr, cannot differentiate structural kidney damage and hemodynamic triggers such as volume depletion (50–52). Other traditional biomarkers of AKI, such as cystatin C, also lack etiological specificity, as they elevate in multiple clinical scenarios (53).

uNGAL can effectively differentiate sustained AKI characterized by nephron damage and significantly higher uNGAL levels (204.8 ± 411.9 ng/dL) from transient AKI, often associated with hemodynamic fluctuations and prerenal azotemia with lower uNGAL (30.8 ± 36.5 ng/dL, similar to non-AKI patients) (54). Bedside uNGAL dipsticks (specificity 91%, negative predictive value 0.97) enable rapid triage by ruling out tubular injury in emergency settings (55).

More emerging evidence demonstrates that uNGAL aids in differentiating AKI etiology and demonstrates distinct uNGAL patterns across AKI subtypes (**Table 2**). Turgut et al. found that patients with postrenal AKI have significantly higher uNGAL levels than prerenal-intrinsic AKI patients (56), while Singer et al. found that uNGAL can effectively distinguish between intrinsic and prerenal AKI and claimed that uNGAL levels over 104 μg/L may be the evidence of intrinsic AKI with a likelihood ratio of 5.97 (57). Similar results were also observed in Seibert et al. and Westhoff et al.’s studies, showing higher uNGAL levels in intrinsic AKI compared with prerenal AKI, with the cut-off values of 52 ng/ml and 654 ng/ml, respectively (58, 59).

**Table 2:** Cut-off values of uNGAL in differential diagnosis of AKI.

Studies	Biomarker	Differentiation	Sample size	Assay type	Age group	Cut-off value	Sensitivity	Specificity	AUC
Turgut, Turk J Med Sci, 2020 (56)	uNGAL	Postrenal AKI vs. prerenal-intrinsic AKI	82	Human Lipocalin-2/NGAL ELISA kits (Biovendor GmbH, RD191102200R)	18–75 years old included	42.625 ng/mL	0.94	0.91	0.957
Singer, Kidney International, 2011 (57)	uNGAL	Intrinsic AKI vs. prerenal AKI	145	ARCHITECT (Abbott Laboratories)	Mean age 67.7 years	104 μg/L	0.75	0.88	0.87
Seibert, Acta Physiol, 2013 (58)	uNGAL	Intrinsic vs. prerenal AKI	87	ELISA NGAL kit 037 (Bioporto)	Mean age 69.6 years	52 ng/mL	0.75	0.72	0.76
Westhoff, Pediatr Nephrol, 2016 (59)	uNGAL	Intrinsic vs. prerenal AKI	141	NGAL Rapid ELISA-Kit (BioPorto Diagnostik)	0–18 years included (classified by pediatric modified RIFLE AKI criteria)	654 ng/mL	0.51	0.93	0.93
Côté, Can J Kidney Health Dis, 2022 (60)	uNGAL	Intrarenal AKI vs. functional AKI	250	NGAL Test™ by BioPorto Diagnostics	Mean age 67.5 years	139 ng/ml	0.84	0.73	0.80
Côté, Can J Kidney Health Dis, 2022 (60)	uNGAL/Cr	Intrarenal AKI vs. functional AKI	250	NGAL Test™ by BioPorto Diagnostics	Mean age 67.5 years	288 ng/mg	0.75	0.80	0.83
Belcher, Hepatology, 2014 (61)	uNGAL	ATN vs. PRA and HRS	188	ELISA methods	Age ≥18 included	365 ng/ml	0.71	–	0.78
Treeprasertsuk, BMC Gastroenterol, 2015 (62)	uNGAL	ATN vs. other AKI	121	ARCHITECT (Abbott Laboratories)	Age ≥18 included	137 ng/mL	0.89	0.80	0.91
Ariza, PLoS One, 2015 (63)	uNGAL/Cr	ATN vs. other AKI	55	Bio-Plex Pro RBM Human Kidney Toxicity panel 2 (Bio-Rad Laboratories)	Mean age 58 years	294 µg/g	0.92	0.89	0.96
Fagundes, Journal of Hepatology, 2012 (64)	uNGAL/Cr	ATN vs. other AKI	241	NGAL ELISA kit (Bioporto)	Mean age 60 years	194 µg/g	0.91	0.82	–
Qasem, ISRN Nephrology, 2014 (65)	uNGAL/Cr	ATN vs. other AKI	160	NGAL ELISA kit (BioVendor GmbH)	Mean age 53.3 years	286 µg/g	0.96	0.76	0.91
Hamdy, Annals of Hepatology, 2018 (66)	uNGAL	Intrinsic AKI vs. HRS	83	NGAL ELISA kit (BioVendor GmbH)	Mean age 54.3 years	143 ng/mL	0.75	0.80	0.82
Barreto, Journal of Hepatology, 2014 (67)	uNGAL/Cr	Persistent AKI vs. transient AKI	65	NGAL ELISA kit (Bioporto)	Mean age 58 years	281 ± 477 vs. 85 ± 79 μg/g	–	–	–
Barreto, Journal of Hepatology, 2014 (67)	uNGAL/Cr	Type-1 HRS vs. other causes of AKI	65	NGAL ELISA kit (Bioporto)	Mean age 58 years	59 ± 46 vs. 429 ± 572 μg/g	–	–	–

AUC, area under the curve; AKI, acute kidney injury; ATN, acute tubular necrosis; PRA, pre-renal azotemia; HRS, hepatorenal syndrome

There are also other studies that supported that uNGAL could be an effective biomarker in differentiating distinct types of AKI, including intrarenal AKI, acute tubular necrosis (ATN, structural tubular cell injury), pre-renal azotemia (PRA, renal function injury caused by decreased renal perfusion without structural renal damage), hepatorenal syndrome (HRS, functional kidney failure associated with advanced liver disease), and so on, despite variability in proposed thresholds (60–67). Côté et al. demonstrated that uNGAL over 139 ng/ml and uNGAL/Cr over 288 ng/mg distinguish intrarenal AKI from functional (prerenal) AKI (60). Numerous studies have proposed cut-off values for distinguishing ATN from other forms of AKI in patients with liver cirrhosis; however, these cut-off values range widely, from 137 to 365 ng/ml (61–65).

Studies of NGAL demonstrate early predictive value in sepsis-induced AKI. Kim et al. observed pNGAL >400 ng/mL in septic AKI versus <200 ng/mL in septic patients without AKI in their meta-analysis (68). Hu et al. reported significantly higher uNGAL levels in patients with septic AKI compared to the septic patients without AKI at 24 h (1081.78 ± 732.65 vs. 450.01 ± 450.79), 48 h (1163.01 ± 853.24 vs. 487.34 ± 520.00), and 72 h (1351.33 ± 796.10 vs. 460.80 ± 442.38) after ICU admission (69). However, differentiating sepsis-induced AKI from other causes remains complex, as systemic inflammation from sepsis independently elevates NGAL levels in the bloodstream and urine, irrespective of kidney injury (70).

Monomeric NGAL has been thought to be a potentially more kidney-specific biomarker for AKI, though its specificity compared to total NGAL assays remains uncertain. Huelin et al. emphasized the significance of monomeric NGAL in identifying different types of AKI in patients hospitalized for decompensated cirrhosis, including hypovolemia-induced AKI (with a history of excessive fluid loss or bleeding within the days before AKI development), HRS-AKI (simultaneous dysfunction of both liver and kidneys), ATN, and miscellaneous AKI, especially differentiating ATN from other types of AKI. Their study showed that the urinary level of monomeric NGAL on day 1 of diagnosis was markedly different across AKI types, and ATN patients had significantly higher values than other types of AKI. The accuracy of uNGAL monomer diagnosing ATN is high with an AUC of 0.80, and the high accuracy persists to day 3 (71). Though theoretically monomeric NGAL is more kidney-specific, its diagnostic accuracy was not superior to total uNGAL in their study.

### 3.3 NGAL’s role in prediction of AKI severity and treatment guidance

The optimal timing for AKI patients to commence KRT remains unclear. Although limited studies showed potential benefit of early KRT, such as the ELAIN trial, which demonstrated reduced 90-d mortality, improved kidney function recovery, shorter duration of mechanical ventilation and hospital stay (72); more randomized controlled studies and meta-analyses demonstrated that early KRT has no beneficial effect on mortality but, on the other hand, may still improve the recovery of kidney function, reduce the length of ICU and hospital stay, and increase the risk of adverse events (73, 74). This underscores the need to refine KRT decision-making, including utilizing reliable biomarkers.

Crucially, NGAL has demonstrated significant predictive power in identifying patients at risk for severe AKI and determining the need for KRT. Emerging evidence suggests that higher NGAL levels may be associated with increased AKI severity, potentially predicting progression to severe outcomes, indicating its possible role in guiding KRT initiation (75–81). Cruz et al. reported that pNGAL levels at ICU admission worked well not only for early diagnosis of AKI (up to 2 days earlier than clinical diagnosis) but also as a predictor for KRT need (AUC 0.82) and correlated with AKI severity (82). Klein et al.’ meta-analysis tested blood NGAL to predict KRT in AKI using data from 22 studies; its sensitivity analysis suggested that a threshold of NGAL>600 ng/ml improves KRT prediction (83), while Senthilkumaran et al. regarded 494 ng/dL as the best cut-off value of pNGAL tested at the ICU admission with high sensitivity and specificity in patients with snakebite-induced AKI (84). Elitok et al. found the uNGAL/hepcidin-25 ratio obtained at induction of anesthesia, within 60 minutes after the end of cardiac surgery, and 1 day after surgery outperformed NGAL to predict AKI-KRT (85).

While NGAL demonstrates predictive capabilities for AKI requiring KRT, its utility as a sole indicator is still challenged by the wide variability in reported cut-off values. Xu et al.’s meta-analysis of 18 observational studies investigated the predictive value of NGAL as a biomarker of AKI requiring KRT in 1,787 of 5,441 patients who developed AKI, the pooled sensitivity and specificity were 0.75 (95% confidence interval, CI: 0.68–0.81) and 0.76 (95% CI: 0.70–0.81), respectively, and its clinical application was still of great value. Their data, however, clearly showed that both plasma/serum and urinary NGAL were predictive of AKI requiring KRT, with cut-off values showing wide variability (1.25 ng/mL to 2600 ng/mL) due to different clinical settings, AKI definitions and NGAL sources, noting that the lack of established cut-off values limits NGAL’s reliability as a sole indicator for guiding KRT initiation (86).

Nevertheless, studies highlight some tangible benefits of NGAL-guided interventions, particularly in optimizing the timing of KRT initiation. Chen et al.’s meta-analysis of 9 randomized controlled trials (RCTs) to investigate the timing of KRT initiation, which suggested that early KRT initiation in the AKI population with high pNGAL can reduce mechanical ventilation days, even though no survival benefit was confirmed, nor was there any difference in the early or late KRT initiation groups for ICU or hospital length of stay, KRT duration, or renal recovery/KRT dependence (87). Despite the increasingly evident utility of NGAL in risk stratification, further robust studies are still needed to fully substantiate the clinical benefits of NGAL-guided intervention. Apart from guiding KRT initiation, in terms of applying a structured biomarker-guided KDIGO−style prevention bundle to reduce the incidence and severity of AKI, there is currently no NGAL-guided AKI “bundle” evidence comparable in strength to the TIMP−2·IGFBP7 trials like PrevAKI (88).

Furthermore, NGAL’s utility extends to providing a broader prognostic assessment, identifying patients at elevated risk for other severe adverse events beyond the need of KRT. NGAL is also effective in predicting mortality, major adverse kidney events to 30 days, and other adverse events, further supporting its value in KRT decision-making in different clinical settings (89–91). In pediatric AKI, urinary NGAL predicted 30-day and 3-month mortality with AUC 0.79 and 0.81 respectively, supporting its good diagnostic performance in pediatric patients with AKI of heterogeneous etiology (89). Among critically ill adults, urinary NGAL improved prediction of progression (adjusted odd ratio 4.6, 95% CI 1.4-17.9), also strongly associated with 30-day KRT or death (90). In emergency department settings, Teo K et al. demonstrated that NGAL predicts both the need for KRT (21% higher risk per 100 ng/dL increase; p < 0.001) and 3-month all-cause mortality (10% higher risk per 100 ng/dL increase; p = 0.028), regardless of existing patient comorbidities (91).

However, the interpretation of NGAL levels is not without its complexities, particularly concerning the distinct roles of monomeric and homodimeric forms. Although monomeric NGAL is considered more kidney-specific and therefore potentially superior for AKI diagnosis, its performance in predicting AKI severity and KRT initiation may be more complicated. Glassford et al. used uNGAL ELISA-1: ELISA-2 ratio >50 as an indicator of monomeric NGAL for ICU patients at risk of AKI and found that patients with detected monomeric NGAL were more likely to develop AKI classified as Injury level (Risk, Injury, Failure, Loss, End stage: RIFLE criteria; generally corresponding to KDIGO Stage 2) and to have septic AKI, liver failure, and elevated pNGAL; while pNGAL (thought to be primarily homodimeric NGAL) showed valuable discrimination for RIFLE-F AKI (corresponding to KDIGO Stage 3) and continuous kidney replacement therapy (CKRT) (92). Namely, monomeric NGAL did not perform better than homodimeric NGAL in the prediction of AKI severity and the need for KRT. It is probably due to sepsis-related homodimeric NGAL production contributing to worse outcomes and increased KRT need, which confounds its predictive specificity. In addition, a previous study reported that the occasional presence of low levels of homodimeric NGAL has minimal impact on the interpretation of total NGAL measurements in urine samples from ICU patients. The investigators employed form-specific immunoassays to quantify monomeric and homodimeric NGAL, alongside a total NGAL assay (93).

### 3.4 Summary and discussion of NGAL in AKI

NGAL is a valuable biomarker for AKI management, throughout early diagnosis, differential diagnosis, and treatment guidance. It responds more rapidly than traditional biomarkers and has the potential to enable earlier diagnosis. NGAL may also differentiate AKI etiologies, including prerenal, intrinsic, and postrenal types, and demonstrates relatively high predictive accuracy (AUC > 0.75) in distinguishing ATN from PRA and HRS. In addition, NGAL levels can indicate AKI severity and predict the need for KRT.

Monomeric NGAL is recognized as a kidney-specific form and may be useful in identifying kidney-specific injuries; therefore, holds bigger potential in early diagnosis of AKI and differential diagnosis of various AKIs, although its effectiveness compared to total NGAL remains to be validated. However, based on limited available data, homodimeric NGAL, but not monomeric NGAL, has been reported to be associated with more severe stages of AKI and CKRT (92). Whether this association is linked to sepsis-related inflammatory effects requires further investigation.

The lack of standardized cut-off values remains a significant challenge in using NGAL as biomarkers in clinical settings, partly attributed to the variable specificity of NGAL forms and the complex etiologies of AKI. Further research is needed to determine appropriate cut-off values, especially if a more specific assay using monomeric NGAL is considered, for example, which might greatly influence its clinical performance and utilization.

## 4 NGAL in peritoneal dialysis‑related peritonitis

Peritonitis is a common and severe complication in peritoneal dialysis (PD) patients (94), may lead to PD technical failure (95). Although improvements have been made in the prevention and treatment of PDRP so that its incident rate has dropped over time (96, 97), its morbidity and mortality are still the main threats to the PD patients, with up to 6.0% mortality and up to 20.4% catheter removal (98). Current diagnostic criteria rely on clinical symptoms/signs, WBC count, and positive cultures (99). However, abdominal pain is absent in some patients with peritonitis (99), the WBC count in peritoneal dialysis effluent (PDE) defined by the International Society for Peritoneal Dialysis (ISPD) still may not be perfect (100, 101), and for certain countries/centers the culture negative rate of peritonitis could be as high as 28%/43%, respectively (94), which leads to the above clinical diagnostic criteria not consistently effective in identifying PDRP.

Some biomarkers, such as matrix metalloproteinase-8 (MMP-8) and interleukin-6 (IL-6) (102–104), MMP-9 (105), and leukocyte esterase (106), have been studied for the diagnosis of PDRP. However, the timeliness and accuracy of diagnosis are still challenging. Even though the matrix metalloproteinase-8 and interleukin-6 (as in the PERiPLEX assay) have shown potential for point-of-care testing (POCT), its insufficient specificity may still need further improvement. Beyond the generic inflammatory nature of IL-6, which can be elevated in both infectious and non-infectious conditions, the potential release of MMP-8 from cells other than neutrophils might contribute to its imperfect specificity too. MMP-8 is not only released by neutrophils but also could be released by various other cell types, including macrophages, plasma cells, T-cells, endothelial cells, smooth muscle cells, oral epithelial cells, fibroblasts, cancer cells, etc. (107–109). Therefore, searching for better novel biomarkers is essential for early diagnosis and treatment monitoring of PDRP.

In recent years, more evidence has become available to support the use of NGAL as a biomarker for early diagnosis and prognosis of PDRP. In bacterial peritonitis, following bacterial invasion, circulating neutrophils migrate into the inflamed peritoneum (110), which will release large amounts of NGAL contributing to the local inflammatory response with its intrinsic role in infection control. NGAL levels in the peritoneal area are significantly upregulated during the infection. Therefore, NGAL, as an infection marker, has been explored for early diagnosis and management of peritonitis. In addition, Leung et al. observed that human peritoneal mesothelial cells can also release NGAL when stimulated by IL-1β (111), which may reverse important events of epithelial-to-mesenchymal transition (EMT) induced by TGF-β during peritonitis. As EMT is pivotal in peritoneal fibrosis and functional impairment in peritoneal dialysis (112), NGAL’s role in PDRP recovery may also be protective.

### 4.1 Diagnostic value of NGAL in PDRP

An increase of plasma NGAL can be observed in a series of infectious conditions such as UTIs, pneumonia, sepsis, etc. (5, 113). In patients with severe acute generalized peritonitis, Axelsson et al. showed a 10-fold increase of pNGAL level compared to the healthy blood donors, correlating with neutrophil activity markers (leukocyte elastase and neutrophil proteinase 4=P3), with increased NGAL in peritoneal exudates too (~40 mg/l) (114).

The different NGAL forms, distinguishable by antibody immunoassays, can provide insights into NGAL’s origin and dynamics; therefore, detecting NGAL forms may aid in early PDRP diagnosis and monitoring antibiotic efficacy. However, most clinical studies to date have not evaluated the diagnostic potential of NGAL forms, particularly the homodimeric form, for PDRP, despite suggestions by Martino et al. that assessing the more specific NGAL forms in peritoneal effluent, which, for example, may help distinguish acute peritonitis from baseline local inflammation, facilitating the use of NGAL in more accurate diagnosis of peritonitis (28). One of the very limited trials to evaluate NGAL forms was to differentiate the NGAL released by neutrophils and/or renal tubular cells in AKI and UTI patients but not in PD-related peritonitis (45).

In 2009, Leung et al. found that in 27 continuous ambulatory peritoneal dialysis (CAPD) patients, NGAL concentrations in serum and PDE increased significantly when they developed peritonitis. PDE NGAL levels were always two to three times higher than the serum NGAL collected on the same day. During acute episodes of peritonitis, NGAL level was strongly correlated with neutrophil count in PDE (first 5 days) and in circulation (first 6 weeks). They also believe that NGAL is not only released by neutrophils but also synthesized in peritoneal mesothelial cells induced specifically by IL-1β (111). Furthermore, the authors indicate that during peritonitis, NGAL released by mesothelial cells may play a protective role in regulating the epithelial-to-mesenchymal transition (EMT) activated after peritonitis. These results suggest both the potential of NGAL as a supplementary diagnostic marker for PDRP and the inherent complexity of its clinical application.

In regard to its baseline condition, Martino et al.’s cross-sectional study involving 69 PD patients showed that the median value of serum NGAL was 487 ng/mL (interquartile range, IQR: 407–586 ng/mL) under basal conditions, in contrast to the poor presence of NGAL in PDE with a median value of 38 ng/mL (IQR: 22.1 - 49.5 ng/mL). It has been proposed due to the limited stimuli to NGAL secretion in the peritoneal cavity in the basal state, as well as the poor diffusion of NGAL from the bloodstream to the peritoneum because of its relatively large molecule size (25,000 Da) (115). Consequently, PDE NGAL shows promise as a biomarker for the detection of peritonitis.

In 2012, Martino et al. evaluated the reliability of PDE NGAL as a diagnostic marker for predicting peritonitis (116). The results revealed significant differences between the case and control groups in serum C-reactive protein (CRP), serum procalcitonin, PDE NGAL, and PDE WBC. Among them, PDE NGAL and PDE WBC count demonstrated the higher AUC for predicting bacterial peritonitis, with AUCs of 1 and 0.99, respectively. They also found that although PDE NGAL was closely associated with PDE WBC count, it appeared to have different behavior. In 6.6% of control group patients, WBC count was elevated (>100 cells/mm^3^), but they had no clinical or microbiological evidence of peritonitis and did not show elevated peritoneal-NGAL. This finding indicates that peritoneal NGAL plays a special role in excluding peritonitis independently of PDE WBC count. At least based on this study, NGAL appears to be a potentially more reliable biomarker than WBC count, particularly in its higher specificity.

In 2015, Martino et al. conducted another larger case-control study over a 19-month period and enrolled 182 PD patients (28). They reported that WBC count and PDE NGAL levels were independent predictors of peritonitis events, PDE NGAL was strongly correlated with PDE WBC count, and the combination of WBC and PDE NGAL could significantly improve the specificity of peritonitis detection, while the concomitant use of WBC or PDE NGAL (at least 1 test positive) increases the sensitivity. The investigators also noted that NGAL-based diagnostic indicators might yield false-negative and false-positive results due to variability in baseline NGAL levels among different PD patients. Therefore, the authors emphasized the importance of distinguishing the local inflammation level during peritonitis from the baseline without peritonitis, for example, potentially by evaluating monomeric and dimeric NGAL in peritoneal effluent.

### 4.2 NGAL for etiologic differentiation in PDRP

The etiology of peritonitis can be classified based on organisms and concomitant events (e.g., tunnel infection) to guide treatment. Gram-positive peritonitis generally has a higher resolution rate compared to gram-negative peritonitis (117), whereas dialysis patients with gram-negative peritonitis experience higher hospitalization rates than those with gram-positive infections (118). Culture-negative peritonitis, where no microorganisms are found in PDE culture, may result from cellular or non-cellular causes of cloudy effluent (119), posing diagnostic and treatment challenges. NGAL may aid in identifying etiology by distinguishing between gram-positive, gram-negative, and culture-negative peritonitis, facilitating diagnosis in patients with suspicious symptoms.

Leung et al. found that NGAL levels in PDE were consistently higher in gram-negative peritonitis than in gram-positive peritonitis, but the difference was not statistically significant. Culture-negative peritonitis displayed lower PDE NGAL levels than both, yet remained approximately 10-fold higher than non-infective PDE (111). Similarly, Lacquaniti et al. reported a significant increase in PDE NGAL levels relative to baseline levels on day 1 of peritonitis onset across all types of peritonitis: gram-positive, gram-negative, or culture-negative (27). Gram-negative bacterial peritonitis showed the highest NGAL level, while negative culture peritonitis showed the lowest, a trend consistent throughout monitoring. Large-scale studies are needed to determine the value of NGAL in identifying the etiology of peritonitis or predicting the clinical outcome of peritonitis.

### 4.3 NGAL point-of-care testing for rapid PDRP diagnosis

The NGAL POCT was developed as a faster alternative to ELISA, which is highly accurate but requires approximately 4 hours for completion (120). In 2022, Virzì et al. evaluated NGAL POCT (NGAL dipstick: NGALds) for PDRP diagnosis, finding strong positive correlations between POCT results and laboratory tests and proposed a cut-off value of 300 ng/mL for peritonitis diagnosis (121); their further study confirmed the efficacy of using NGAL POCT for early PDRP detection and diagnosis (122). With a 20-minute testing time, NGAL POCT is a rapid, user-friendly tool for early PDRP diagnosis.

In addition, a more recent multicenter prospective observational study in China enrolled 221 PD patients (42 peritonitis and 179 non-peritonitis). They demonstrated that a homodimeric NGAL rapid test kit is a highly effective tool for diagnosing PDRP with sensitivity and specificity rates of 100% and 99.44%, respectively, showing exceptional promise as a reliable and rapid diagnostic tool for PDRP (123). This should be the first clinical trial to verify a more specific NGAL form (homodimeric NGAL) in PDRP diagnosis.

For better understanding of its performance, especially its sensitivity and any influence of time and temperature, PDE samples from 18 PDRP patients (from home and hospital settings) in Thailand were all tested positive by the same homodimeric NGAL rapid test kit, based on the data presented during the 62^nd^ European Renal Association (ERA) congress in 2025 (124). The median WBC count was 1,260 (592, 4,604) cells/µL. After serial dilution to determine the lowest neutrophil count detectable by this POCT test, the median minimum neutrophil counts yielding a positive result at time zero and room temperature (RT) was 8.50 (5.00-19.25) cells/µL, indicating very high sensitivity. Within the mixed testing ranges (T0, 1-hour, 2-hour, 12-hour, and RT, 35, 40, and 45 °C), the median neutrophil counts range from 6 to 16/µL.

Lastly, Prieto-Magallanes et al. performed a meta-analysis to investigate the diagnostic accuracy of measuring NGAL in peritoneal/ascitic fluid in spontaneous bacterial peritonitis (SBP) and PDRP (125). Of the ten studies with quantitative data, four of them were studies in patients with PDRP from diverse geographic settings (Egypt, Italy, China, and the United States); the pooled accuracy reported: sensitivity of 0.96 (95% CI, 0.88–0.99), specificity of 0.88 (95% CI, 0.50–0.98), and AUC of 0.94.

### 4.4 NGAL for treatment monitoring and outcome prediction in PDRP

The monitoring of NGAL post peritonitis can potentially deliver valuable information about the therapeutic responses and peritoneal function. Leung et al. identified a correlation between serum NGAL levels and peripheral blood neutrophil count post-peritonitis, with both increasing significantly on day 1 (103). Similarly, NGAL in PDE peaked on day 1 post-peritonitis and remained high for the next two days and was significantly correlated with neutrophil count in PDE on days 1, 2, 3, and 5. NGAL in all PDE specimens declined rapidly from day 5 after the onset of peritonitis, correlating with clinical response to antibiotic treatment and neutrophil count in PDE.

Lacquaniti et al. conducted a 12-month study of 30 patients undergoing CAPD (27). The results showed that PDE WBC gradually decreased after antibiotic treatment in all tested peritonitis subgroups and showed a statistically significant decrease on day 4 ± 0.6. In contrast, PDE NGAL showed a statistically significant reduction on day 3 ± 0.4 after peritonitis onset, roughly one day earlier than PDE WBC. Furthermore, in patients with a positive response to treatment, PDE WBC returned to normal levels around 7 days after peritonitis, while PDE NGAL took 23 ± 4 days to return to normal, which may be related to NGAL secretion by peritoneal mesothelial cells during peritonitis. Notably, this study also showed that NGAL had high sensitivity (88.8%) and specificity (90.4%) in identifying empirical antibiotic treatment failure 3 days after the diagnosis of peritonitis and was superior to WBC count (sensitivity 77.8%, specificity 57.1%).

In addition, a more recent study conducted by Virzì et al. measured PDE WBC count and PDE NGAL on days 0, 5, 10, and 15 after the onset of peritonitis in 22 patients with PDRP and observed a parallel decline in WBCs and NGAL levels in PDE during the course of peritonitis in patients with favorable outcomes; statistically significant higher levels were observed in both PDE WBC count and NGAL on days 10 and 15 in patients with negative outcomes, with a non-significant trend toward elevated levels on day 5 (29). Furthermore, during peritonitis monitoring, patients who exhibited a sudden rise in PDE WBCs also demonstrated a significant corresponding increase in PDE NGAL. These data suggest the potential value for PDE NGAL as an outcome predictor in PDRP patients.

### 4.5 Summary and discussion of NGAL in PDRP

Although NGAL has demonstrated value in the diagnosis and treatment monitoring of PDRP, including potential superiority over some other biomarkers, as well as possible contribution to shedding light on the etiology of PDRP, it has not yet been widely adopted in clinical practice. Further studies are warranted to evaluate its performance using reliable and reproducible assays, ideally with larger sample sizes, and in alignment with ISPD guidelines, to compare against the current standard of care.

Unlike the earlier studies, more recent studies on NGAL POCT offer renewed promise for earlier detection and diagnosis, particularly if implemented more broadly in home settings. As demonstrated in the PROMPT study, each hour of delay in PDRP treatment is associated with a 5.5% increase in treatment failure (126). While preliminary findings on the use of homodimeric NGAL POCT in PDRP patients have shown promising sensitivity and specificity, further research is needed to validate its effectiveness in home-based applications, assess its ability to reduce the time from symptom onset to treatment, and evaluate its impact on clinical outcomes, including treatment failure rates.

## 5 Conclusion

NGAL has firmly established itself as a pivotal early biomarker for AKI, offering a significant advantage over traditional markers like serum creatinine by enabling detection within hours of injury, apart from its great value in risk stratification, severity prediction and potential biomarker-guided clinical intervention in AKI. In PDRP, NGAL in the dialysis effluent demonstrates comparable, if not superior, diagnostic potential for early infection detection. The diagnostic performance of NGAL, particularly the homodimeric form, shows promise for point-of-care testing, which could significantly improve clinical efficiency. However, the full clinical translation of NGAL in both areas is hampered by challenges such as the lack of standardized, form-specific assays and the confounding influence of systemic inflammation, which affects specificity.

To enhance diagnostic precision, some future efforts must focus on differentiating NGAL forms, as emphasized by others too (28, 41, 127). Monomeric NGAL, originating from renal tubular cells, is a more specific indicator of kidney damage, while homodimeric NGAL derives from neutrophils. Validating assays that can distinguish these forms is critical for differentiating true kidney injury from systemic inflammation in AKI and for providing more timely and accurate diagnosis in PDRP. Furthermore, establishing standardized, population-specific cut-off values through large-scale, multicenter studies is essential to address the current variability and make NGAL a more reliable tool across diverse clinical settings.

Despite its promise for AKI and PDRP management, current evidence on NGAL is constrained by small sample sizes, heterogeneous populations, and confounding factors such as inflammation and chronic kidney disease, which may reduce specificity. Assay variability and lack of standardized cut-offs further limit reproducibility, while more specific molecular forms remain largely inaccessible for routine use. Moreover, this review is narrative in nature and does not apply a more robust systematic literature search and analysis typically used in systematic reviews, which may introduce selection bias. These limitations highlight the need for standardized assays, larger multicenter studies, improved assay availability for more specific forms of NGAL, and future, more rigorous data to strengthen evidence and optimize NGAL’s integration into clinical practice.

In summary, realizing the full potential of NGAL to improve patient outcomes in AKI and PDRP hinges on bridging the translational gap. This requires a concerted effort to advance assay technology, conduct robust clinical validation, and achieve widespread standardization. By doing so, NGAL can evolve from a predictive biomarker into a guiding tool for personalized clinical decision-making.

## Future perspectives

The evolving landscape of NGAL as a biomarker underscores the need for molecular form-specific advancements to overcome current limitations in AKI and PDRP management. Very limited form-specific NGAL trials have been attempted in other settings as well, such as kidney transplantation (4), yet commercially available form-specific NGAL assays remain largely scarce.

While total NGAL is clinically useful despite lacking standardized cut-offs, monomeric NGAL—with its renal tubular origin—holds substantial potential for precise differential diagnosis in AKI subtypes, particularly in sepsis-associated AKI (SA-AKI), where systemic inflammation confounds total NGAL. Research priorities must include validating its role in subtype differentiation and optimizing the timing of KRT. Looking ahead, integrating NGAL forms into AI-driven multi-biomarker panels—alongside other reliable biomarkers, such as tissue inhibitor of metalloproteinases-2/insulin-like growth factor binding protein 7 (TIMP-2/IGFBP7), and/or clinical variables—could enable bedside risk scoring and personalized therapeutic guidance in critical care.

In PDRP, defining kinetic profiles of NGAL forms (especially homodimeric) throughout infection and treatment phases may refine diagnosis and enable real-time monitoring—offering an alternative to the current rather pragmatic ISPD WBC threshold (100, 101). The most critical next step is establishing rapid, cost-effective POCTs for clinical and, more importantly, home use—devices that could reduce PDRP treatment delays and failure rates by 5.5% per hour (PROMPT study (126)). Large-scale, multicenter studies are needed to validate these tools in real-world settings.

Collaborative efforts between academia, industry, and regulators are essential to deliver standardized kits for clinical use. NGAL has the unique potential to anchor precision nephrology, shifting care from reactive to predictive and transforming patient outcomes.

## Glossary

ADPKD: Autosomal Dominant Polycystic Kidney Disease
ADQI: Acute Disease Quality Initiative
AKI: Acute Kidney Injury
ATN: Acute Tubular Necrosis
AUC: Area Under the Receiver-Operating Characteristic Curve
BNP: B-Type Natriuretic Peptide
BUN: Blood Urea Nitrogen
CAPD: Continuous Ambulatory Peritoneal Dialysis
CI: Confidence Interval
CKD: Chronic Kidney Disease
CKRT: Continuous Kidney Replacement Therapy
CRP: C-Reactive Protein
EMT: Epithelial-to-Mesenchymal Transition
ERA: European Renal Association
HRS: Hepatorenal Syndrome
IGFBP7: Insulin-like Growth Factor Binding Protein 7
IL-6: Interleukin-6
IQR: Interquartile Range
ISPD: International Society for Peritoneal Dialysis
KDIGO: Kidney Disease: Improving Global Outcomes
KRT: Kidney Replacement Therapy
MMP-8: Matrix Metalloproteinase-8
MMP-9: Matrix Metalloproteinase-9
NGAL: Neutrophil Gelatinase-Associated Lipocalin
NGALds: NGAL Dipstick
PD: Peritoneal Dialysis
PDE: Peritoneal Dialysis Effluent
PDRP: Peritoneal Dialysis-Related Peritonitis
pNGAL: Plasma NGAL
PRA: Pre-Renal Azotemia
POCT: Point-of-Care Testing
PRA: Prerenal Azotemia
RCTs: Randomized Controlled Trials
RT: Room Temperature
SA-AKI: Sepsis-Associated AKI
SBP: Spontaneous Bacterial Peritonitis
SCr: Serum Creatinine
SLE: Systemic Lupus Erythematosus
TIMP-2: Tissue Inhibitor of Metalloproteinases-2
TGF-β: Transforming Growth Factor-β
uNGAL: Urinary NGAL
UTIs: Urinary Tract Infections
WBC: White Blood Cell.
